# CRISPR-directed epigenetic reprogramming of the FLT1 locus: a novel strategy for reversing fetal hemoglobin silencing in β-thalassemia minor

**DOI:** 10.1097/MS9.0000000000004847

**Published:** 2026-03-31

**Authors:** Maheen Naz, Maryam Batool, Syed Abdullah Shah, Muhammad Talha, Mahnoor Fatima

**Affiliations:** a Department of Medicine, Isra University, Karachi, Pakistan; b Department of Medicine, Rahbar Medical and Dental College, Lahore, Pakistan; c Department of Medicine, King Edward Medical University, Lahore, Pakistan; d Department of Medicine, Shaheed Ziaur Rahman Medical College, Rajshahi Medical University, Bogura, Bangladesh

*To the Editor,*

β-Thalassemia is an inherited hemoglobin disorder caused by reduced or absent β-globin synthesis, resulting in ineffective erythropoiesis and chronic anemia. It has been estimated that 1.5% of the world’s population is carriers of β-thalassemia trait. People with β-thalassemia minor carry a change (mutation) in just one of the two β-globin genes. They may have mild anemia, so their hemoglobin level is lower than normal, usually around 9–11 g/dL^[^1^]^. Current clinical management primarily includes regular blood transfusions coupled with iron chelation therapy to manage iron overload, yet these treatments are associated with complications such as transfusion dependency, organ toxicity, and a substantial healthcare burden. Hematopoietic stem cell transplantation offers a potential cure, but it is linked with transplant-related morbidity, uncertain donor availability, and immunological incompatibility^[^2^]^. At the molecular level, inadequate adult hemoglobin (HbA) production highlights the therapeutic relevance of fetal hemoglobin (HbF) reactivation, which can partially compensate for β-globin deficiency and improve red blood cell survival^[^3^]^. Consequently, CRISPR-based platforms have introduced transformative therapeutic approaches to treat β-thalassemia minor, a safe and durable HbF-inducing strategy. This letter is in line with the TITAN Guidelines on the need for transparency in AI use in healthcare^[^4^]^.

CRISPR therapy includes CRISPR/Cas9, base editors, and prime editors. CRISPR–Cas9 genome editing has emerged as a powerful approach for correcting β-hemoglobinopathies by modulating globin gene regulation. CRISPR-Cas9–based therapy is a genome engineering approach that enables sequence-specific targeting of regulatory elements controlling globin gene expression. In β-thalassemia, therapeutic benefit is achieved not by correcting β-globin mutations directly, but by reactivating HbF, which can functionally compensate for deficient HbA. CRISPR-mediated disruption of HbF repressors or their regulatory enhancers, most notably within erythroid-specific transcriptional networks, results in sustained γ-globin expression and increased HbF production in erythroid cells. This hemoglobin switching strategy improves red cell survival and ameliorates ineffective erythropoiesis without altering the underlying β-globin genotype^[^5^]^. In β-thalassemia minor, even modest HbF induction may be sufficient to improve hemoglobin balance and clinical symptoms.

Despite the success of BCL11A-focused approaches, not all patients show the same increase in HBF, which means other genes also influence HbF levels. Although BCL11A is a major repressor of HbF, targeting BCL11A alone does not guarantee consistent or generalizable HbF reactivation. The CRISPR-Cas9 study of the BCL11A erythroid enhancer reports outcomes in only two patients and explicitly acknowledges limitations, including lack of clonal diversity analysis and uncertainty regarding long-term stem cell behavior and broader applicability^[^6^]^. Analysis of Cameroonian and Tanzanian cohorts identified FLT1 as a novel locus associated with HbF variance. FLT1 significant variants occurred at higher frequencies (minor allele frequency 0.076–0.105) and accounted for approximately 3–3.5% of HbF variation. Fine mapping indicated that the likely causal variants reside near the FLT1 promoter and a candidate enhancer, many within transcription factor binding sites involved in erythropoiesis. Individuals carrying the minor alleles exhibited higher HbF levels, supporting a functional role for these variants in HbF regulation^[^7^]^. These findings indicate FLT1 as an important new genetic contributor to HbF variation in African populations with β-thalassemia.

Despite its therapeutic promise, CRISPR-Cas9 genome editing faces several critical limitations, particularly in human hematopoietic stem and progenitor cells, which demonstrated that editing outcomes are strongly influenced by the timing and regulation of DNA repair pathways, with nonhomologous end joining frequently generating heterogeneous and unpredictable edits. Inefficient or uncontrolled DNA repair can reduce precise editing efficiency and compromise genomic integrity, posing risks such as unintended insertions, deletions, or chromosomal abnormalities^[^8^]^. A primary concern in clinical gene editing is the potential for the off target effects, which could lead to unintended genomic alterations and oncogenic risks. In addition, the high cost of gene editing therapies, often exceeding $1 million per patient, poses a significant barrier to equitable access^[^2^]^.

In conclusion, while BCL11A remains a central target for HbF reactivation, its biological and safety limitations highlight the need for complementary approaches. FLT1-directed epigenetic modulation represents a promising, mechanistically distinct, and ethically favorable strategy for achieving safer and more consistent HbF induction in β-thalassemia minor. Further functional validation and long-term studies are warranted to assess its clinical potential.

## Data Availability

Not applicable to this study.
